# Glomangioma of Uncertain Malignant Potential: A Case Report

**DOI:** 10.1155/2020/4237076

**Published:** 2020-01-08

**Authors:** Meghan McCullough, Bonnie Balzer, Stuart H. Kuschner

**Affiliations:** ^1^Division of Plastic Surgery, Keck School of Medicine, University of Southern California, Los Angeles, CA, USA; ^2^Anatomic Pathology, Musculoskeletal Pathology Service, Cedars Sinai Medical Center, Los Angeles, CA, USA; ^3^Department of Orthopedics, Cedars Sinai Medical Center, Los Angeles, CA, USA

## Abstract

Glomus tumors are rare benign tumors which commonly affect the hand but are seldom seen extradigitally. Less commonly seen is the glomangioma, a variant of benign glomus tumor, and even rarer is the glomangiosarcoma, a malignant variant. Determining malignancy can be difficult and an intermediate diagnosis, glomus tumor of uncertain malignant potential, has been proposed. We present a case of a 56-year-old male with a recurrent forearm mass diagnosed as a glomangioma of uncertain malignant potential. Although the characteristics and behavior of malignant cases are still incompletely understood, it is important that a high index of suspicion be maintained when approaching these tumors, especially when large or recurrent. Glomangiomas should be included in the differential diagnosis when evaluating soft tissue masses in the forearm and should be evaluated for malignant features.

## 1. Introduction

Glomus tumors are rare benign tumors which commonly affect the hand but are seldom seen in other areas [1]. They arise from the glomus body, a neuromyoarterial structure that controls blood pressure and temperature through the blood flow in the skin [2]. Less commonly seen is the glomangioma, a variant of benign glomus tumors, histologically characterized by an abundance of vascular structures [3]. Even rarer is the glomangiosarcoma, a malignant glomus tumor. Deciding which glomus tumors are benign and which ones are malignant is not always easy. In 2001, Folpe et al. reviewed the criteria for a diagnosis of malignancy and proposed an intermediate diagnosis: glomus tumor of uncertain malignant potential [4].

We present a case of a 56-year-old male with a recurrent forearm mass diagnosed as a glomangioma of uncertain malignant potential. Glomangiomas should be included in the differential diagnosis when evaluating soft tissue masses in the forearm, and one should be aware that glomangiomas can have malignant features.

## 2. Case Presentation

**Figure 1:**
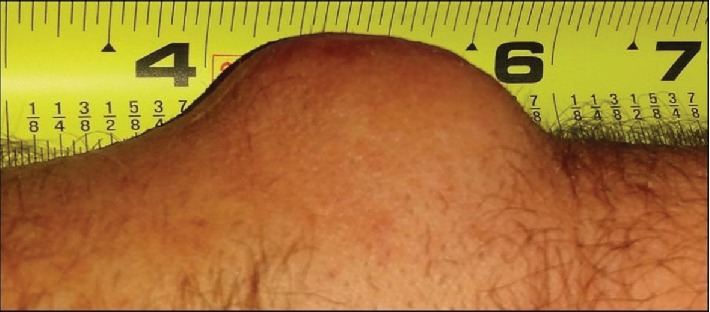
Photographs provided by the patient from 2015, prior to surgical re-excision.

**Figure 2:**
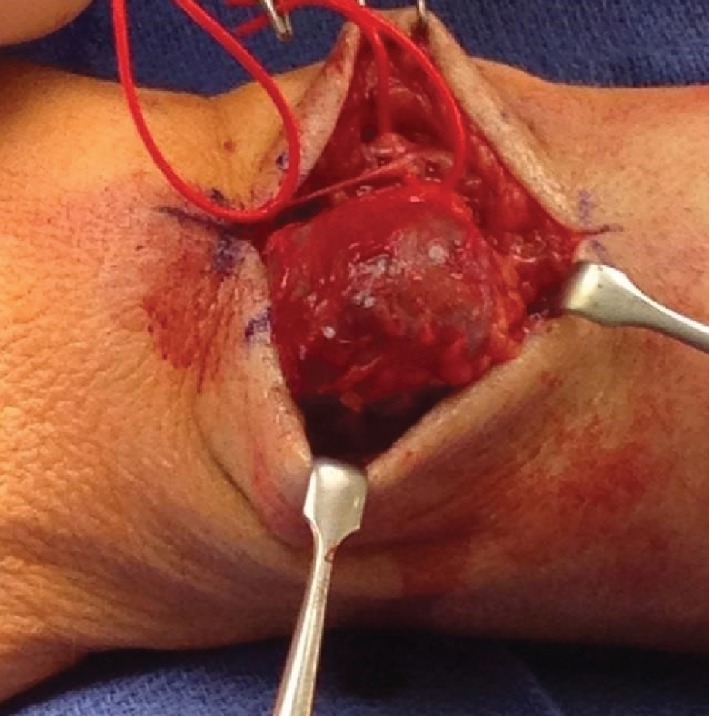
Intraoperative excision.

**Figure 3:**
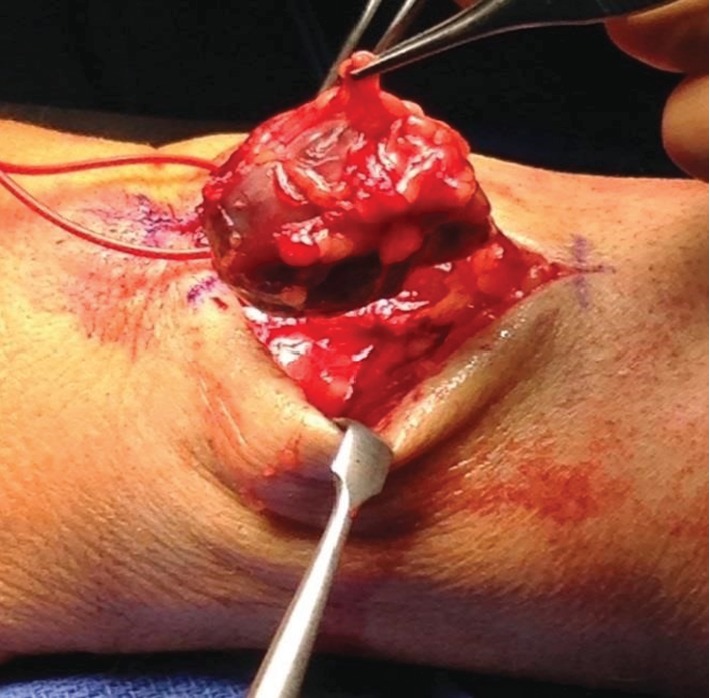
Intraoperative excision.

The authors have obtained the patient's informed written consent for print and electronic publication of the case report. The patient is a 56-year-old male who first noticed a mass on the dorsum of his left distal forearm in 1993. He recalls that the mass was generally painless but the “slightest external pressure” resulted in “instantaneous pain of great intensity.” He did not seek medical attention, and the mass gradually enlarged. In 1999, he was seen by an orthopedic surgeon who diagnosed a ganglion cyst and attempted aspiration. No fluid was obtained. Shortly thereafter, he was seen by a vascular surgeon who resected what preoperatively was felt to be a vascular hemangioma. However, the pathological examination was reported as glomangioma. After surgery, the mass was no longer visible but his symptoms did not abate. He continued to have exquisite pain with palpation over the surgical site. Over time, a mass recurred. He was first seen in our office in 2010, at which time, there was a 2.5 cm diameter mass at the dorsum of the left distal forearm. Surgery was scheduled and then cancelled by the patient who did not return until April 2015 at which time the mass had grown to 5 cm in diameter, still painful to palpation. He underwent excision of the mass in November 2015.  Figure 1 is a photograph taken shortly before surgery in 2015. Figures 2 and 3 are photographs taken at the time of surgery.

**Figure 4:**
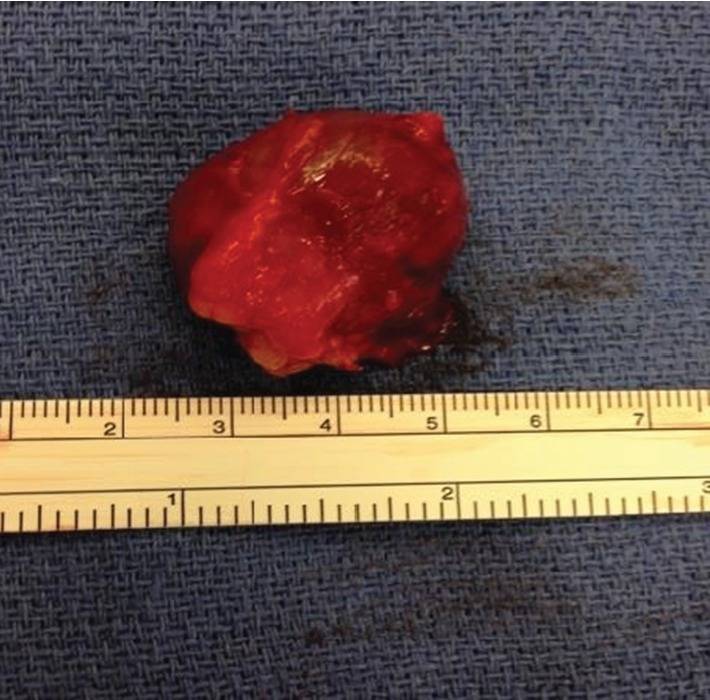
Specimen after excision.

**Figure 5:**
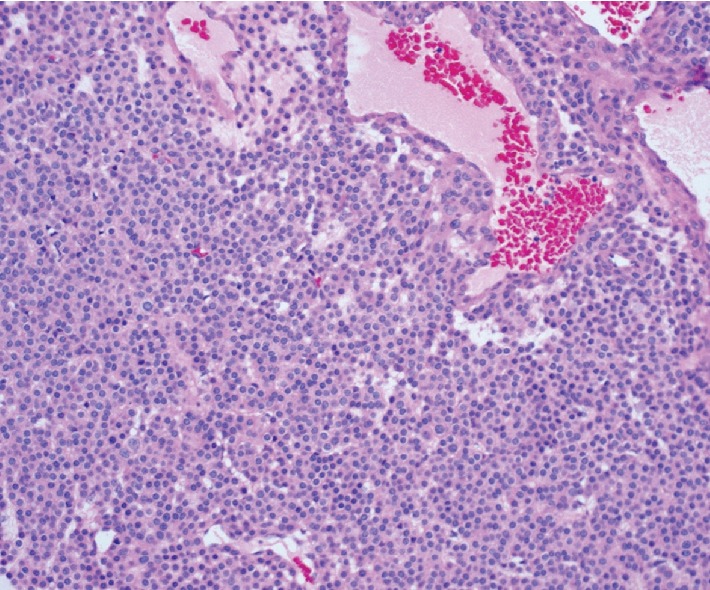
Medium power view of the glomus tumor cells showing round nuclei, relatively even chromatin, and eosinophilic cytoplasm with distinct nuclear borders, arranged around small-to-large ectatic vascular channels.

**Figure 6:**
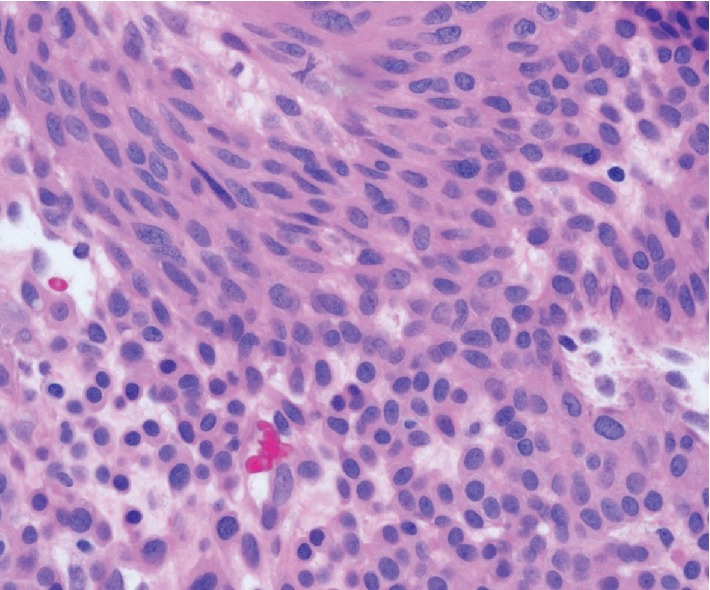
Transition between conventional appearing rounded glomus tumor cells and more spindled glomangiomyomatous cells at the top of the photograph, which are in a perivascular distribution (high power).

Figure 4 shows the specimen sent to pathology and Figures 5 and 6 show the histologic slide. The specimen was diagnosed by the reviewing pathologist (B.B.) as a glomangioma of uncertain malignant potential. It measured 3.3 × 2.7 × 2.0 cm, superficial to the fascia, with a mitotic index of <1 MF/50 HPF (less than 1 mitotic figure per high-power field), and without evidence of necrosis or atypia. The large size and recurrence were felt to be the criteria concerning for aggressive behavior. Other criteria for malignancy—deep location, atypical mitotic figures, moderate-to-high nuclear grade, and high mitotic index (5+/50 HPF)—were all lacking.

Unlike after his first operation, the patient experienced complete resolution of his symptoms after excision of the mass. At 4-year follow-up, there is no evidence of recurrence of disease.

## 3. Discussion

A glomus body is a neuromyoarterial body found within the reticular dermis that functions as a specialized form of arteriovenous anastomosis and is responsible for thermoregulation. Wood first described a glomus tumor in 1812 as a painful subcutaneous nodule made worse by changes in temperature and cured by surgical removal [5]; the histopathologic characteristics were originally reported in 1924 by Masson [6]. Overall, they represent 1-2% of soft tissue tumors [7]. Extradigital sites reported include the palm, wrist, forearm, foot, bone, stomach, colon, trachea, vagina, cervix, and mesentery [7, 8].

Multiple attempts have been made to categorize tumors comprised of glomus cells including solitary, multiple, solid, diffuse, adult, and pediatric. More recently, these tumors have been simplified into two major subtypes: the glomus tumor and the glomangioma [9]; however, disagreement still exists over the exact definition of these terms. The term glomangioma was coined by Monteagudo in 1935 for lesions with wide vascular lumens, which are most commonly found in patients with multiple tumors [10].

While many authors use the term glomangioma to describe multifocal lesions, in fact, the two entities show different clinical, etiological, and histopathologic features. The glomus variants are small, painful, and purple nodules with predilection for acral areas of the extremities, especially the nail beds of the fingers and toes [11]. Aching pain, well-localized tenderness, and temperature sensitivity are the characteristic triad of signs and symptoms [7]. In contrast, glomangiomas have an angiomatous appearance to the lesions. Glomangiomas often appear during adolescence as small pink to bluish nodules situated deep in the dermis and may be widely scattered. They are rarely subungual and are less likely to be painful [11–13].

Glomus tumors arise as sporadic tumors, and while glomangiomas may also be sporadic, autosomal-dominant inheritance patterns with incomplete penetrance, and variable expressions have been described [14–18]. Familial glomangiomas have been mapped to chromosome 1p21-p22 and are thought to be a result of loss of function mutations in the cytoplasmic protein glomulin [19, 20].

Histopathologically, glomus tumors contain vascular channels surrounded by glomus cells. The glomus cells are monomorphic round or polygonal cells with large nuclei and scant eosinophilic cytoplasm. In contrast, glomangiomas contain more dilated venous channels than glomus tumors and resemble venous malformations. However, unlike venous malformations, they demonstrate single-to-multiple rows of surrounding cuboidal glomus cells [12, 19, 21].

Glomangioma is the most common variant of glomus tumor and shows a more vascularized rather than solid growth pattern compared to glomus tumor. About 1% of glomus tumors and glomangiomas are reported to be malignant [22]. In superficial locations such as this one (above the fascia), malignant glomus tumors are exceedingly rare. In 1990, Gould et al. made the first attempt to characterize malignancy [23]. In 2001, Folpe et al. identified the criteria for malignancy based on a series of 52 cases. Criteria for establishing a diagnosis of malignant glomus tumor and glomus tumor (or variant) of uncertain malignant potential are large size (>2.0 cm) and deep location or moderate-to-high nuclear grade, and increased mitotic rate (>5 per 50 high-power fields) or the presence of atypical mitotic figures. [4]. If these histologic criteria of malignancy are met, the risk of metastases exceeds 25%. However, tumors with some but not all of these features, such as this case (size > 2.0 cm), are best described as glomus tumors with uncertain malignant potential. Our patient had already shown a local recurrence. It should be noted that the possibility of incomplete resection cannot be completely ignored even though the mass was no longer visible to the patient. However, regardless of the margins on the previous excision, most glomus tumors show only a small risk of recurrence.

The recurrence and metastatic potential of glomangiosarcoma has not been fully elucidated secondary to a paucity of cases in the literature. Variants of glomus tumor, including glomangiomyoma and glomangiomyopericytoma and symplastic glomus tumor, all are considered benign unless they exhibit the criteria for aggressive behavior listed above.

## 4. Conclusion

Although glomus tumors and glomangiomas are a well-recognized cause of pain in the digits, they are often overlooked when formulating the differential diagnosis of extradigital lesions. Because extradigital tumors are more difficult to diagnose, patients often suffer from delayed diagnosis and/or misdiagnosis. According to several studies, the average duration of symptoms is reported to be between 7 and 11 years [24–26] and patients will undergo 2.5 consultations before diagnosis [26]. This finding is similar to other reports of atypical location glomus tumors, in which the diagnosis was not obtained for 5 to 20 years [27–29].

While rare, glomus tumors and glomangiomas can be malignant. Although the characteristics and behavior of malignant cases are still incompletely understood, it is important that a high index of suspicion be maintained when approaching these tumors, especially when large or recurrent. Patients may present to a diverse group of physicians, including dermatologists, plastic surgeons, general surgeons, orthopedists, and pain specialists. It is important that all these health professionals maintain glomangioma in the differential diagnosis to ensure speedy diagnosis and treatment, as well as to be aware that there is a malignant variant.
